# Protective and Anti-Inflammatory Effects of Protegrin-1 on *Citrobacter rodentium* Intestinal Infection in Mice

**DOI:** 10.3390/ijms22179494

**Published:** 2021-08-31

**Authors:** Celina Osakowicz, Lauren Fletcher, Jeff L. Caswell, Julang Li

**Affiliations:** 1 Department of Animal Biosciences, Ontario Agricultural College, University of Guelph, Guelph, ON N1G 2W1, Canada; cosakowi@alumni.uoguelph.ca (C.O.); lfletc03@uoguelph.ca (L.F.); jcaswell@uoguelph.ca (J.L.C.); 2 Department of Pathobiology, Ontario Veterinary College, University of Guelph, Guelph, ON N1G 2W1, Canada

**Keywords:** antimicrobial peptide, protegrin-1, immunomodulatory effect, *Citrobacter rodentium* infection, infectious colitis

## Abstract

Infectious intestinal colitis, manifesting as intestinal inflammation, diarrhea, and epithelial barrier disruption, affects millions of humans worldwide and, without effective treatment, can result in death. In addition to this, the significant rise in antibiotic-resistant bacteria poses an urgent need for alternative anti-infection therapies for the treatment of intestinal disorders. Antimicrobial peptides (AMPs) are potential therapies that have broad-spectrum antimicrobial activity due to their (1) unique mode of action, (2) broad-spectrum antimicrobial activity, and (3) protective role in GI tract maintenance. Protegrin-1 (PG-1) is an AMP of pig origin that was previously shown to reduce the pathological effects of chemically induced digestive tract inflammation (colitis) and to modulate immune responses and tissue repair. This study aimed to extend these findings by investigating the protective effects of PG-1 on pathogen-induced colitis in an infection study over a 10-day experimental period. The oral administration of PG-1 reduced *Citrobacter rodentium* intestinal infection in mice as evidenced by reduced histopathologic change in the colon, prevention of body weight loss, milder clinical signs of disease, and more effective clearance of bacterial infection relative to challenged phosphate-buffered saline (PBS)-treated mice. Additionally, PG-1 treatment altered the expression of various inflammatory mediators during infection, which may act to resolve inflammation and re-establish intestinal homeostasis. PG-1 administered in its mature form was more effective relative to the pro-form (ProPG-1). To our knowledge, this is the first study demonstrating the protective effects of PG-1 on infectious colitis.

## 1. Introduction

Infectious intestinal colitis caused by bacteria, viruses, or parasites is a widespread condition affecting millions of humans worldwide each year [1,2]. With pathophysiology including diarrhea, intestinal inflammation, and epithelial barrier disruption, bacterial intestinal colitis contributes a significant portion of total acute diarrhea cases, which resulted in 1.3 million deaths globally in 2015 [1]. Antibiotic therapy is a common treatment; however, the rising threat of antibiotic resistance presents a problem for the effective treatment and clearance of the infection [1]. The pathogenesis of these disorders is thought to involve genetic susceptibility and abnormal immune responses to environmental factors or intestinal bacteria, which result in uncontrolled intestinal inflammation [3]. The high prevalence of infectious intestinal colitis in humans and the significant rise of antibiotic-resistant bacteria has warranted an urgent need for alternative anti-infective therapies.

Antimicrobial peptides (AMPs) are a potentially valuable therapy for infectious intestinal colitis and intestinal disorders. These small cationic and amphipathic molecules constitute an initial mechanism of host defense in both prokaryotes and eukaryotes, with a broad spectrum of antimicrobial activity against bacteria, fungi, enveloped viruses, and parasites [4,5,6,7]. AMPs have functions beyond microbial killing and immune modulation, such as angiogenesis, vascularization, wound healing, and tumor surveillance [6,8,9,10]. AMP expression is dysregulated in the inflamed intestine of Crohn’s disease and ulcerative colitis patients, and this may be a significant factor in the loss of mucosal maintenance; however, their full contributions have yet to be identified [11,12,13].

Cathelicidins are characterized by a conserved N-terminal cathelin domain capable of inhibiting the cathepsin-L protease [14] and may be involved in immune modulation (reviewed in [15]). Protegrins are an important class of cathelicidins expressed in porcine leukocytes [16]. Five highly homologous protegrins have been described (PG-1 to PG-5) [17]. Naturally produced by neutrophils, they are arginine- and cysteine-rich and assume a rigid β-hairpin structure stabilized by two disulphide bridges [18]. Protegrins maintain their antimicrobial activity in physiological salt solutions, enhancing their therapeutic potential in intestinal disorders, as these conditions resemble those found in extracellular fluids and serum [19]. Of the protegrins, PG-1 is considered as one of the most potent AMPs: its activity has been widely demonstrated in vitro and in vivo, and it can eliminate multi-drug-resistant microorganisms [18]. PG-1 has been shown to protect mice from the intraperitoneal and intravenous challenges of multiple types of bacteria, suggesting potential for PG-1 in the treatment of local or systemic infections [18,20]. In a chemically induced mouse model of colitis, ProPG-1 decreased pro-inflammatory tumor necrosis factor alpha (TNF) and cyclooxygenase-2 (COX-2) expression while increasing that of epithelial tissue repair trefoil factor 3 (TFF3), providing evidence of its ability to modulate inflammation and enhance tissue repair [21]. However, the effect of PG-1 in an infectious colitis model has not yet been investigated. This infection study aimed to investigate the effects of ProPG-1 and mature PG on bacterial load, inflammation, and indicators of disease using a *C. rodentium*-induced colitis mouse model over a 10-day trial period.

## 2. Results

### 2.1. PG-1 Reduced C. rodentium—Induced Intestinal Infection

**Figure 1:**
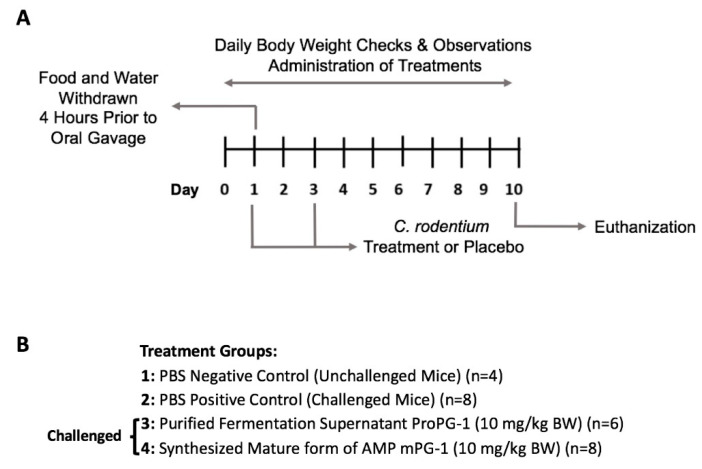
Animal Study Design. (**A**) The trial timeline for induction of colitis. (**B**) Treatment groups. Mice were challenged with *C. rodentium* (2 × 10^9^ CFU/mL) or left unchallenged (PBS) and were treated daily with ProPG-1, mPG-1, or PBS. Two mice within the ProPG-1 group died due to gavage complications; six mice in this group completed the study.

**Figure 2:**
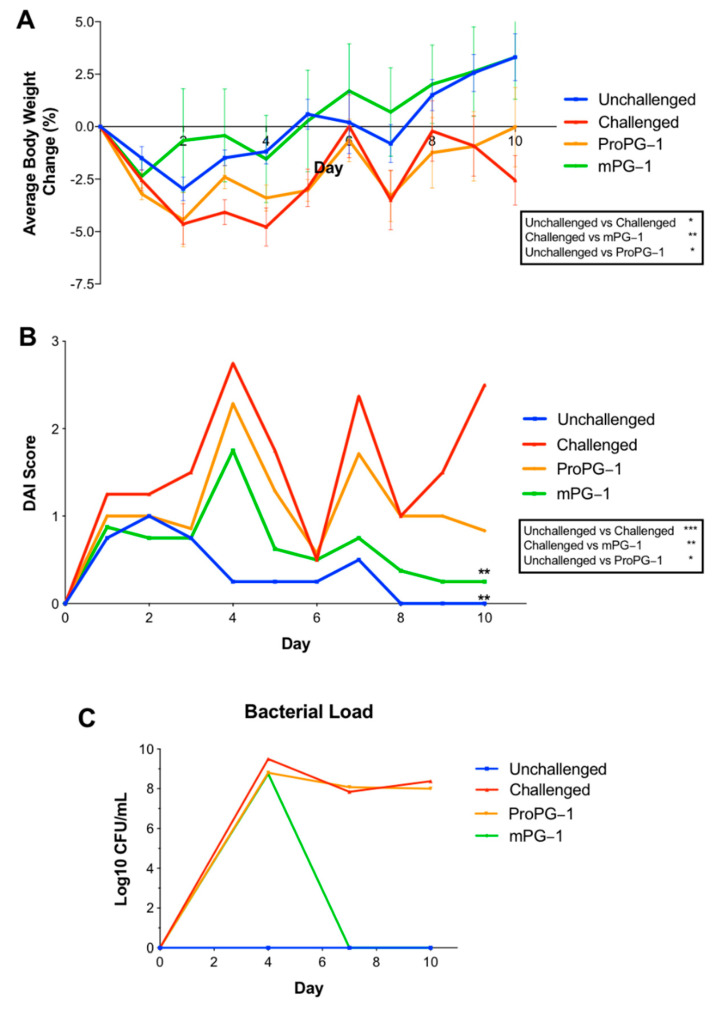
Effects of PG-1 on body weight, Disease Activity Index (DAI), and bacterial load. Protegrin treatments reduced body weight loss (**A**), DAI (**B**), and *C. rodentium* infection (**C**) compared to challenged mice receiving PBS. A higher DAI score represents more severe disease signs. ** *p* < 0.01 indicates DAI on day 10 is significantly different than in challenged mice receiving PBS. Body weight and DAI data represent the mean ± SEM; * *p* < 0.05, ** *p* < 0.01, *** *p* < 0.001 calculated by a one-way repeated measures ANOVA post hoc Tukey test over the course of the trial. Bacterial colony counts are expressed as Log_10_ CFU/mL.

The animal study design is outlined in Figure 1. Significant weight loss (*p* < 0.05; Figure 2A) and increased DAI (disease activity index) scores (more severe disease signs) (*p* < 0.001; Figure 2B) were present over the course of the trial in the *C. rodentium*-challenged mouse group compared to unchallenged mice. All treatment groups had slightly increased DAI scores on Days 1, 2, and 3 compared to Day 0, likely attributed to food and water starvation for the first dose of *C. rodentium* and/or to initial stress from gavage. All *C. rodentium*-challenged groups had increased DAI scores beginning on Day 4, while unchallenged mice returned to levels similar to Day 0. Visually, *C. rodentium*-challenged mice displayed ruffled coats, lower levels of activity, and moist stool/diarrhea. All mice survived until endpoint, except for 2 mice that died due to gavage complications.

mPG-1 treatment inhibited *C. rodentium*-induced body weight loss over the course of the trial (*p* < 0.01; Figure 2A). mPG-1-treated *C. rodentium*-challenged mice had similar weight gains as unchallenged mice and 5.9% greater weight gains on Day 10 compared to challenged PBS-treated mice, although this was not statistically significant (*p* = 0.06; Figure 2A). Similarly, the DAI of mPG-1-treated *C. rodentium*-challenged mice initially increased but subsequently decreased over the course of the trial (*p* < 0.01), and the DAI was 10-fold lower on Day 10 relative to that of *C. rodentium*-challenged mice treated with PBS (*p* < 0.01) and comparable (*p* = 0.74) to the DAI of unchallenged mice (Figure 2B).

*C. rodentium*-challenged proPG-1-treated mice, relative to challenged PBS-treated mice, had slightly reduced but statistically insignificant body weight loss (Figure 2A) and DAI scores (Figure 2B) from Day 1 until Day 8. After Day 8, challenged ProPG-1-treated mice began to increase in body weight, returning to their starting body weight on Day 10, with lower DAI scores compared to challenged PBS-treated mice, although the difference was statistically insignificant (Figure 2A,B).

*C. rodentium* was not detected in any fecal samples from unchallenged mice for the duration of the trial, and all other treatment groups had similar bacterial loads (average of 9.0 log_10_ CFU/mL) by Day 4 (Figure 2C). While both *C. rodentium*-challenged proPG-1-treated and PBS-treated groups had an average bacterial load of 8.0 log_10_ CFU/mL on Day 7 and Day 10, *C. rodentium* was not detected in the *C. rodentium* challenged mPG-1-treated group on either of these days. By Day 10 post-infection, both the unchallenged and the *C. rodentium*-challenged mPG-1-treated groups had undetectable fecal water content, whereas the *C. rodentium*-challenged ProPG-1-treated group had 3.03% fecal water content, less than the 5% fecal water content measured for the *C. rodentium*-challenged group treated with PBS (data not shown). No significant differences were observed in the length or weight of the small intestine, colon, and cecum among all treatment groups (data not shown).

### 2.2. PG-1 Decreased C. rodentium—Induced Histologic Lesions in the Colon

**Figure 3:**
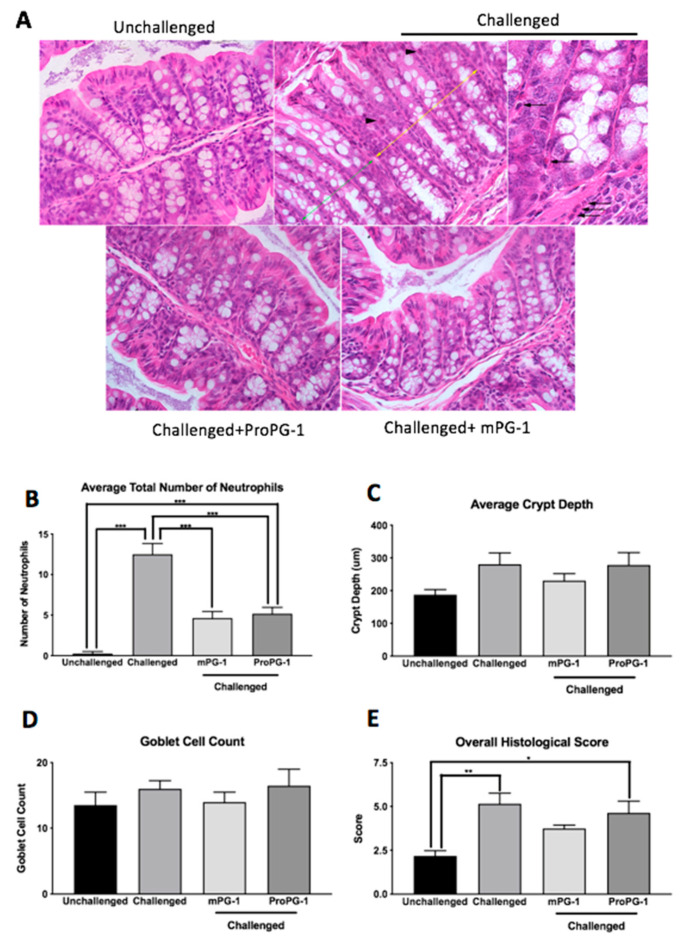
Effects of PG-1 on histologic lesions (**A**) and colonic histomorphology (**B**–**E**). Distal and proximal colonic sections on Day 10 showed altered mucosal morphology in *C. rodentium-*challenged mice (upper center and upper right images [enlarged]) compared to unchallenged mice (upper left image). The challenged mice had crypt hyperplasia with loss of goblet cells (yellow arrow–lesional area; green arrow–normal area), necrosis of individual enterocytes (arrowhead), and infiltration of neutrophils (arrows). Both ProPG-1- and mPG-1-treated challenged mice (lower left and right images, respectively) had milder lesions than PBS-treated mice, but mPG-1-treated challenged mice had less immune cell infiltration. Representative hematoxylin-and-eosin-stained sections of colon (magnification: 400×). Compared to untreated mice challenged with *C. rodentium*, both ProPG-1- and mPG-1-treated challenged mice had a decrease in (**B**) average total number of neutrophils. No significant differences were observed among treatments for (**C**) average crypt depth or (**D**) goblet cell counts, but both ProPG-1- and mPG-1-treated challenged mice showed a decrease in overall histologic score (**E**). Data represent the mean ± SEM; * *p* < 0.05, ** *p* < 0.01, *** *p* < 0.001 compared to controls; one-way ANOVA post hoc Tukey test.

Colonic crypt hyperplasia, loss of goblet cells, necrosis of enterocytes, and immune cell infiltration were observed in H&E-stained sections of colon from mice challenged with *C. rodentium*, whereas unchallenged mice showed no significant lesions (Figure 3A). The administration of ProPG-1 and mPG-1 resulted in milder mucosal lesions in challenged mice, and mPG-1 treatment resulted in less immune cell infiltration in comparison to challenged, untreated mice (Figure 3A).

Neutrophil counts were significantly lower in ProPG-1- and mPG-1-treated *C. rodentium*-challenged mice compared to *C. rodentium*-challenged PBS-treated mice (*p* < 0.001; Figure 3B). Similar trends were observed for crypt depth and goblet cell count, although the differences between groups were not statistically significant (Figure 3C,D). No significant differences in lamina propria width or tunica muscularis width were found among the treatment groups. Compared to unchallenged mice, overall histological scores (calculated as per Table S1) were increased in *C. rodentium*-challenged PBS-treated (*p* < 0.01) and *C. rodentium*-challenged ProPG-1-treated (*p* < 0.05) groups, while treatment with mPG-1 in *C. rodentium*-challenged mice reduced the histological score to a level that was not significantly different from that of the unchallenged group (Figure 3E).

### 2.3. PG-1 Modulates Intestinal Gene Expression during C. rodentium Infection

**Figure 4:**
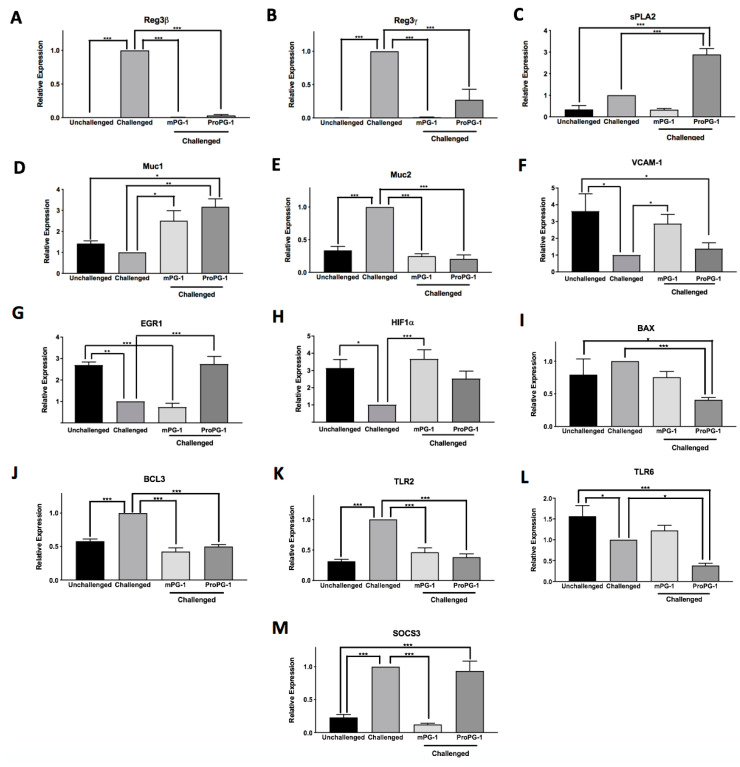
Effects of PG-1 on antimicrobial peptide (AMP) gene expression (**A**–**C**), self-protecting gene expression (**D**–**H**), and apoptotic, innate immune response, and cytokine gene expression (**H**–**M**) within the colon. Protegrin treatments differentially affect gene expression of the AMPs Reg3β (**A**), Reg3γ (**B**), and sPLA2 (**C**), Muc1 (**D**), Muc2 (**E**), cell adhesion molecule VCAM-1 (**F**), cell proliferation gene EGR1 (**G**), stress response factor HIF1α (**H**), apoptotic markers BAX (**I**) and BCL3 (**J**), innate immune response genes TLR2 (**L**) and TLR6 (**L**), and the cytokine regulator SOCS3 (**M**). Colonic gene expression levels were determined by RT-qPCR, with values normalized against GAPDH and β-actin. Data represent the mean ± SEM; * *p* < 0.05, ** *p* < 0.01, *** *p* < 0.001 compared to controls calculated by a one-way ANOVA post hoc Tukey test. All values in gene expression analysis were compared to those of challenged mice set to a relative expression of 1.

The expression of epithelial regenerating islet-derived proteins 3β and 3γ (Reg3β and Reg3γ) was markedly up-regulated in *C. rodentium*-challenged PBS-treated mice compared to unchallenged mice, and this induced expression was suppressed in the challenged mPG-1-treated and *C. rodentium*-challenged ProPG-1-treated mice (*p* < 0.001; Figure 4A,B). No difference was observed in the expression of secretory phospholipase A2 (sPLA2) among the groups of unchallenged, *C. rodentium*-challenged, and *C. rodentium*-challenged mPG-1-treated mice. However, treatment with ProPG-1 in *C. rodentium*-challenged mice resulted in an increase (*p* < 0.001) in sPLA2 expression compared to *C. rodentium*-challenged PBS-treated and unchallenged mice (Figure 4C).

Mucin-1 (Muc1) expression was increased by 2.5-fold in *C. rodentium*-challenged mPG-1-treated (*p* < 0.05) and 3.2-fold in *C. rodentium*-challenged ProPG-1-treated groups (*p* < 0.01) compared to the *C. rodentium*-challenged PBS-treated group. The opposite was observed for mucin-2 (Muc2) expression, which was significantly decreased in *C. rodentium*-challenged mPG-1-treated and *C. rodentium*-challenged ProPG-1-treated groups to a level comparable to that in unchallenged mice (Figure 4D,E). The expression of vascular cell adhesion molecule 1 (VCAM-1) was significantly suppressed by *C. rodentium* challenge, and this suppression was completely reversed by mPG-1 treatment but not by ProPG-1 treatment (Figure 4F). mPG-1 did not have any significant effects on early growth response 1 (EGR1) expression compared to PBS treatment in *C. rodentium*-challenged mice, whereas ProPG-1 treatment increased EGR1 expression level to that of the unchallenged control (*p* < 0.001; Figure 4G). Additionally, *C. rodentium* infection suppressed the expression of stress-response hypoxia inducible factor 1 α subunit (HIF1α), which mPG-1 treatment significantly reversed, while ProPG-1 treatment had no significant effect on its expresion compared to all other treatments and conditions (Figure 4H).

No difference in the expression of the apoptotic regulator BCL-2-Associated X (BAX) was observed among the unchallenged, *C. rodentium*-challenged PBS-treated, and *C. rodentium*-challenged mPG-1-treated mice, although its level was down-regulated by ProPG-1 treatment (Figure 4I). The expression of anti-apoptotic B cell leukemia/lymphoma 3 (BCL3) was up-regulated by *C. rodentium* infection, but this was reversed to levels comparable to those in the unchallenged control by both mPG-1 and ProPG-1 treatments (*p* < 0.001; Figure 4J). The expression of Toll-like receptor 2 (TLR2) was significantly up-regulated by *C. rodentium* infection but was down-regulated by mPG-1 and ProPG-1 to the levels observed in unchallenged mice (*p* < 0.001; Figure 4K). Treatment of mPG-1 had no effect on Toll-like receptor 6 (TLR6) expression relative to either of the controls, although its expression was suppressed in the *C. rodentium*-challenged ProPG-1-treated group compared to both *C. rodentium*-challenged and unchallenged groups (Figure 4L). Lastly, *C. rodentium* induced the expression of the suppressor of cytokine signaling 3 (SOCS3), which was reversed by mPG-1 treatment but not by ProPG-1 treatment to levels comparable to those in unchallenged mice (Figure 4M).

## 3. Discussion

The murine-specific bacterium *C. rodentium* is a common model of enteric bacterial infection as it induces intestinal inflammation (colitis). A dosage of 2 × 10^9^ CFU/mL was selected based on preliminary trials conducted and consistently with former studies, as this dosage has been standardized for this infection model [22,23]. Consistent with previous research, C57BL/6 mice orally challenged with *C. rodentium* displayed transient weight loss and diarrhea [24]. The in vivo antimicrobial role of PG-1 was first identified in a study that challenged mice with *Pseudomonas aeruginosa*. In that study, a single IP injection of PG-1 decreased the mortality rate to 0–27%, compared to that of untreated mice, which reported 93–100% mortality [18]. Additionally, transgenic mice ectopically expressing PG-1 had enhanced resistance to *Actinobacillus suis* infection resulting in an 87% survival rate in comparison to a 37% survival rate of wild-type mice [20]. In a mouse model of colitis, all three protegrin domains (ProPG-1, cathelin, and mature PG) partially prevented body weight loss and improved overall DAI scores in comparison to a challenged control group [21]. The present study extended this finding and revealed that oral administration of mPG-1 decreased *C. rodentium*-induced body weight loss and diarrhea and reduced or eliminated *C. rodentium* bacterial load. These results demonstrate that protegrin treatments, especially mPG-1, help to reduce the infection characteristics of colitis and are capable of clearing *C. rodentium* infection. To our knowledge, this is the first study showing the protective effects of PG-1 on pathogen-induced colitis.

A histological hallmark of *C. rodentium* infection is intestinal crypt hyperplasia, which can result from intestinal inflammation and damage, triggering epithelial cell proliferation visible as crypt elongation [25,26]. In our study, *C. rodentium* infection increased overall histological scores compared to the unchallenged control mice, while mPG-1 treatment reduced this histological change. This is consistent with a previous finding in the DSS mouse model of colitis, in which recombinant ProPG-1 and mPG-1 supplementation improved histomorphological changes with a decrease in mucosal epithelial damage and inflammation [21]. Interestingly, this previous study reported that ProPG-1 and mPG-1 treatment increased goblet cell counts by 47% and 53%, respectively, compared to DSS. However, no reduction in goblet cell numbers was observed in the current study, regardless of infection or treatments. It appears that this cellular response to *C. rodentium* is not significant.

Infection with *C. rodentium* triggers a robust host inflammatory response including the expression of various cytokines, AMPs, and innate immune response genes [27]. Our RT-qPCR analysis results generally suggest that PG-1 modulated the pathogen-induced intestinal expression of these genes. Reg3β and Reg3γ are expressed by intestinal epithelial cells upon innate recognition of bacterial components through TLRs and play a role in bacterial killing [28]. High levels of Reg3 proteins have been described in the rodent ileal mucosa and feces during Salmonella infection, increasing with the severity of infection [29]. The gene expression of both Reg3β and Reg3γ were significantly decreased in *C. rodentium*-challenged ProPG-1- and *C. rodentium*-challenged mPG-1-treated mice compared to *C. rodentium*-challenged PBS-treated mice, perhaps due to a decreased level of *C. rodentium* infection. sPLA2, an AMP with pro-inflammatory properties, is expressed in colonic epithelial and goblet cells and is secreted into the intestinal lumen to promote the synthesis of prostaglandins, which aids in the recovery of damaged tissue during inflammation [30]. Our finding that *C. rodentium*-challenged ProPG-1-treated mice had an increase in sPLA2 gene expression compared to *C. rodentium*-challenged PBS-treated mice suggests that ProPG-1 may modulate inflammation during infection to enhance tissue repair. Together with the observation that *C. rodentium* was cleared in mPG-1-treated mice by Day 7, it is possible that the *C. rodentium*-challenged mPG-1-treated mice did not display an increase in sPLA2 gene expression relative to *C. rodentium*-challenged PBS-treated mice due to reduced tissue damage and less need for tissue repair factors.

Muc1 and Muc2 are O-glycosylated proteins that create a mucus layer coating the gastrointestinal tract to protect the epithelium from direct bacterial interactions [31,32]. In our study, *C. rodentium*-challenged mPG-1- and *C. rodentium*-challenged ProPG-1-treated mice showed increased *Muc1* gene expression relative to *C. rodentium*-challenged PBS-treated mice, suggesting enhanced protection from the infection. This agrees with a previous colitis study that demonstrated than the absence of Muc1 leads to the intensification of chronic inflammation [33] and body weight loss [34]. These findings may explain why *C. rodentium*-challenged protegrin-treated mice had reduced body weight loss compared to *C. rodentium*-challenged PBS-treated mice. Muc2 has been shown to protect against lethal colitis infections by binding to and removing bacteria that accumulate on the bacterial surface [35]. This is consistent with our observation that *C. rodentium*-challenged mice showed an increase in Muc2 expression relative to unchallenged mice, suggesting the host increases mucus production to help rid the mucosal surface of the pathogen. Interestingly, our data showed that *C. rodentium*-induced Muc2 expression was downregulated in both challenged mPG-1- and challenged ProPG-1-treated mice to a level comparable to that in unchallenged mice. A reduction of Muc2 expression by protegrin-1, possibly via a negative-feedback loop triggered by excessive mucus production, may be necessary to reduce mucus hypersecretion, a characteristic of chronic inflammatory diseases involving mucosal surfaces [36].

Inflammation is a host’s response to infection and tissue injury, involving the complex coordination of many diverse mediators and regulatory pathways for protection from infection and restoration of homeostasis [37]. Adhesion molecules such as VCAM-1 play a role in inflammation by facilitating the adhesion of leukocytes at the site of infection to help regulate inflammation and stimulate tissue healing [38,39]. *C. rodentium*-challenged mPG-1-treated mice showed increased VCAM-1 gene expression relative to *C. rodentium*-challenged PBS-treated mice, suggesting a role in the stimulation or recruitment of inflammatory cells for intestinal repair. Interestingly, *C. rodentium*-challenged ProPG-1-treated mice did not display increased VCAM-1 expression. However, differential expression may solely be part of the commencement of leucocyte migration [40], as by Day 10, the level of infection in *C. rodentium*-challenged mPG-1- and *C. rodentium*-challenged ProPG-1-treated mice appeared to be different. Moreover, EGR1, a gene involved in cell proliferation [41], can be induced by stress signals such as injury [42]. *C. rodentium*-challenged ProPG-1-treated mice showed upregulated EGR1 expression relative to *C. rodentium*-challenged PBS-treated mice and comparable expression with respect to the unchallenged control. In response to bacterial invasion, colonic epithelial cells undergo controlled apoptosis to remove infected or injured cells, allowing for increased cell proliferation of the epithelial cells of the intestinal lining to restore epithelial integrity [43]. Therefore, the up-regulation of EGR1 in the *C. rodentium*-challenged ProPG-1-treated group suggests that it may be working towards epithelial restoration. Interestingly, *C. rodentium*-challenged mPG-1-treated mice did not display upregulated EGR1 expression, possibly due to mPG-1 clearing the infection by this time point, resulting in reduced apoptosis and less need for cell proliferative factors. HIF1α is a stress response gene vital for the maintenance of intestinal homeostasis by increasing the expression of protective mucosal barrier genes and initiating defensive innate immune responses [44]. The increased expression of HIF1α in *C. rodentium*-challenged protegrin-treated groups compared to *C. rodentium*-challenged PBS-treated mice further suggests PG-1 may play a role in enhancing host protection from bacterial infection, which is also reflected at the physiological level.

Apoptosis is a vital component in the regulation of immune responses during inflammation. BAX, a major pro-apoptotic factor, can be induced by a variety of apoptotic stimuli [45] and can be regulated directly or indirectly by many factors/mediators [46]. The downregulation of BAX in *C. rodentium*-challenged ProPG-1-treated mice but not in *C. rodentium*-challenged mPG-1-treated mice suggests that ProPG-1 may play a role in pathways that regulate BAX to reduce cell death and intestinal sloughing, while *C. rodentium*-challenged mPG-1-treated mice may no longer need to inhibit bacterial-induced apoptosis, as they had effectively cleared infection by this time. Contrastingly, Bcl-3, a pro-survival/anti-apoptotic factor [47], was up-regulated in *C. rodentium*-challenged mice compared to unchallenged mice, and *C. rodentium*-challenged mPG-1- and ProPG-1-treated mice had levels similar to those of the unchallenged PBS-treated group. Increased expression in the challenged group may be a result of the host’s response to prevent excessive cell death caused by bacterial infection by promoting cell survival [47]. It is possible that the protegrin treatments assisted in combating infection, and thus Bcl-3 expression remained similar to that in the unchallenged mice. Consequently, a balance of apoptotic regulators seems critical for re-establishing intestinal homeostasis during inflammation.

TLR2 is up-regulated in bacterial infections, as its agonists are microbial cell wall components, and its stimulation activates innate immune response cascades to eliminate pathogens [48]. Consistent with our results, *C. rodentium*-challenged PBS-treated mice displayed an up-regulation of TLR2 gene expression, while *C. rodentium*-challenged mPG-1- and *C. rodentium*-challenged ProPG-1-treated mice showed a down-regulation, matching the unchallenged mice gene expression levels. This suggests that PG-1 may have the ability to suppress *C. rodentium*-induced immune stimulation and counteract the overstimulation of cytokines which have been shown to induce tissue damage and intestinal barrier destruction [49]. Contrastingly, TLR6 gene expression was up-regulated in unchallenged mice compared to *C. rodentium*-challenged PBS-treated mice, and while mPG-1treatment had no effect on TLR6 gene expression, ProPG-1 treatment appeared to further suppress the expression of the receptor in *C. rodentium*-challenged ProPG-1-treated mice relative to both *C. rodentium*-challenged and unchallenged mice. These results are conflicting, as TLR6 has been shown to be up-regulated in the colon during colitis, stimulating T helper 1 and 17 (Th1/Th17) responses to influence the severity of disease [50]. However, the role of TLR6 on mucosal surfaces is still not well understood, and a consensus yet to be established.

The regulation of cytokines is also an important part of the innate immune response to inflammation. SOCS3 is a negative regulator of cytokines, interfering with cytokine signaling and regulating downstream signaling by other cytokines [51]. The upregulation of SOCS3 gene expression level in *C. rodentium*-challenged PBS-treated mice and *C. rodentium*-challenged ProPG-1-treated mice may be due to various cytokines being stimulated under inflammatory conditions, which in turn induces SOCS3 expression and cytokine level regulation. While the ProPG-1 treatment was less effective than the mPG-1 treatment, mPG-1-treated mice reduced SOCS3 gene expression level to the level in unchallenged mice, suggesting less inflammation.

Taken together, our results suggest that PG-1 can influence the expression of various AMPs, cytokines, and inflammatory mediators which help modulate inflammation, resolve intestinal infection, and work towards reestablishing homeostasis, as demonstrated at the physiological, histological, and gene expression level. However, ProPG-1-treatment appeared to be less effective than mPG-1-treatment. This may be attributed to AMPs’ direct bacterial killing ability, which appears critical in infection control. *C. rodentium*-challenged mPG-1-treated mice effectively cleared infection, while *C. rodentium*-challenged ProPG-1-treated mice remained infected, attempting to re-establish intestinal homeostasis. ProPG-1 treatment may be less effective than mPG-1 due to inefficient cleavage between the cathelin and the antimicrobial mPG-1 domains via the enterokinase (EK) recognition site. The mPG-1 domain must be separated from the cathelin domain to initiate microbicidal action [52], and a low cleavage efficiency could limit its bacterial killing capability. EK protease does not exhibit stringent specificity for the DDDDK sequence, as it has been found that it can preferentially cleave at more accessible external off-target sites [53]. The addition of denaturants, such as urea, may be beneficial for cleavage, as it has been shown to enhance EK specificity [53]. Inefficient cleavage could also stem from the upregulation of protease inhibitors in intestinal infections [54]. Despite inefficient cleavage, the ProPG-1-treatment remained effective in some respects, as ProPG-1 displayed similar gene expression modulation of Reg3β, Reg3γ, Muc1, Muc2, Bcl3, and TLR2, to mPG-1. Although not statistically significant, ProPG-1 also displayed similar trends to mPG-1, with a reduction in BW loss, DAI scores, and fecal water content. These effects in modulating infection could be due to the released mPG-1 or the tissue repair function of ProPG-1, as shown previously [21], or to both in combination. Future studies should further examine the potential influence of Pro-PG-1 and mPG-1 on the host’s microbiota. This study was limited technically by the volume of fecal sample collected from each mouse, sufficient to perform bacterial colony counts to confirm *C. rodentium* infection establishment, but not sufficient to complete the microbiota analysis.

*C. rodentium* is known to share similarities with other attaching–effacing human pathogens such as enteropathogenic *E. coli* (EPEC) and enterohemorrhagic *E. coli* (EHEC) and is extensively used to replicate intestinal disorders such as bacterial intestinal colitis and ulcerative colitis [1,55]. Using a *C. rodentium* challenge model, our study demonstrated the protective effects of PG-1 on intestinal infection via its antimicrobial activity, immunomodulatory effects, and tissue repair capabilities. Its ability to stimulate the regulation of inflammation, enhance intestinal protection and repair, and ultimately clear infection suggests that this AMP could potentially serve a therapeutic use in combating colitis.

## 4. Materials and Methods

### 4.1. Production of Recombinant ProPG-1 in Large-Scale Bioreactor Fermentations

Construction of the *Pichia pastoris* (*P. pastoris*) ProPG-1 expression vector and transformation into *Escherichia coli DH5* utilizing the heat-shock method was performed as previously described [56], with the modification of using a glyceraldehyde-3-phosphate dehydrogenase (GAP) promoter in place of the alcohol oxidase (AOX1) promoter. Purified digested ProPG-1 fragments were ligated into the pD915 *P. pastoris* expression vector from ATUM (Newark, CA, USA) with the GAP promoter using T4 DNA Ligase (Promega; Madison, WI, USA), per supplier’s instructions. Subsequent transformation into *P. pastoris* competent wild-type X33 (Invitrogen; Waltham, MA, USA) by electroporation was performed as previously described [56]. Transformants were screened for protein expression level, and the strain possessing the highest recombinant ProPG-1 expression was selected for large-scale fermentation.

Large-scale fermentations for recombinant ProPG-1 production were conducted in a bioreactor (BioFlo/CelliGen 115 Benchtop Bioreactor, New Brunswick Scientific, Edison, NJ, USA) as outlined previously, with slight modifications [56]. Bioreactor temperature was reduced from 30 °C to 26 °C at 24 h fermentation time to further stimulate protein expression and secretion of recombinant ProPG-1. Additionally, due to the use of the GAP promoter, a 50% (*v*/*v*) glycerol (Thermo Fisher Scientific, Waltham, MA, USA) feed was applied at 24 h fermentation time and maintained at 15 mL/L/h until 48 h fermentation time, and then the fermentation was concluded, and a methanol feed step was not performed. Cultures were centrifuged for 1 h at 4 °C at 3000 rpm to collect the recombinant ProPG-1-containing supernatant. Supernatants were stored at −20 °C for downstream applications.

### 4.2. Protein Sample Preparation

At approximately 95% purity, standard synthetic lyophilized mature PG-1 (Top-Peptide Corporation; Shanghai, China) was dissolved in filter-sterilized 0.01% acetic acid supplemented with 0.1% bovine serum albumin (BSA) (Sigma Aldrich, St. Louis, MO, USA) to decrease non-specific plasticware adsorption, and diluted to working-stock concentrations of 200 ng/µL. Standard mature PG-1 was stored at −80 °C until use. Recombinant ProPG-1 from fermentation supernatant was dialyzed against 25 mM Tris-Cl (pH 7.0) and concentrated through ultracentrifugation using an Amicon^®^ Ultra-15 10 kDa cut-off centrifugal filter (Millipore Sigma; Darmstadt, Germany) per the manufacturer’s instructions. Ultracentrifugation retentate and supernatant flow-through were filter-sterilized and desalted with 1 x phosphate-buffered saline (PBS), then stored at −80 °C.

### 4.3. Ethics Statement

All experiments and procedures involving the use of animals were approved by the University of Guelph Animal Care Committee (AUP #3250) in accordance with the Canadian Council on Animal Care guidelines.

### 4.4. Bacterial Preparation

*C. rodentium* DBS 100 (provided by Dr. B. Coombs (McMaster University, Burlington, ON, Canada)) was cultured in LB broth at 37 °C at 200 rpm overnight before being harvested at 4000× *g* for 10 min at room temperature. Pelleted bacteria were washed and resuspended in sterile 1× PBS at a concentration of 2.0 × 10^9^ colony-forming units (CFU) per mL. The viable CFU counts of inocula were determined by retrospective plating on MacConkey agar plates (24 h at 37 °C).

### 4.5. Animals and Induction of Colitis

*C. rodentium* is a commonly used to obtain a model of colitis to investigate enteric bacterial infections and observe host–pathogen interactions in vivo [55,57,58]. Two pilot animal studies were conducted in C57BL/6 mice to determine the optimal dosage and dosage timepoints of 2.0 × 10^9^ CFU/mL through oral gavage of *C. rodentium* on Day 1 and Day 3 for induction of intestinal inflammation (data not shown).

Six- to eight-week-old C57BL/6 female mice were purchased from Charles River Laboratories (St. Constant, QC, Canada). All mice were housed under temperature-controlled Level 2 Biosafety conditions in the Isolation Facility at the University of Guelph. After acclimatization, mice were fasted for 4 h prior to initial infection (Day 1), when mice were administered 2.0 × 10^9^ CFU/mL of *C. rodentium* DBS 100 via orogastric gavage. A second dose was given on Day 3 or Day 5 to ensure pathogen establishment. Unchallenged mice were orally gavaged with 150 uL of PBS instead of *C. rodentium*. Administration of recombinant ProPG-1 (10 mg/kg body weight), synthetic mPG-1 (10 mg/kg body weight), or PBS was performed on Day 0 and once daily for the duration of the trial. Mice were weighed and scored for Disease Activity Index (DAI) daily. Mice were euthanized via CO_2_ asphyxiation on Day 8 or Day 10.

### 4.6. Measurement of Bacterial Load and Fecal Water Content

To determine bacterial load in the stool, fresh fecal pellets were collected throughout the study. Fecal samples were diluted to 0.1 g of feces per mL in sterile 1× PBS. Samples were homogenized via vortex and serially diluted onto MacConkey agar plates to determine CFU/mL. Viable bacteria were determined as previously described [26,59] and counted after 24 h incubation at 37 °C.

As a measurement of diarrhea within the stool, fresh fecal pellets from individual cages were collected shortly before euthanasia (Day 8 or Day 10). Samples were weighed, incubated for 24 h at 60 °C, and weighed again. The percentage of fecal water content was calculated by dividing the difference of wet and dry weight by the wet weight [60].

### 4.7. Disease Activity Index Score

Animals were assessed daily to determine their disease activity index (DAI) score as previously published by Maxwell [61] and assess the severity of disease. The DAI score was determined as the sum of scores for stool consistency, stool blood, and body weight loss (modified from [61], Table S2). Percent body weight loss of the mice in relation to their initial body weight on Day 0 was calculated using the following formula [61]:

$$\%\;\mathrm{Body}\;\mathrm{Weight}\;\mathrm{Loss}=\frac{\left(\mathrm{Body}\;\mathrm{Weight}\;\mathrm{on}\;\mathrm{Day}\;X-\mathrm{Initial}\;\mathrm{Body}\;\mathrm{Weight}\right)}{\mathrm{Initial}\;\mathrm{Body}\;\mathrm{Weight}}\times 100$$

### 4.8. Tissue Collection

Immediately following euthanasia, the small and large intestines were removed, separated, weighed, and straightened for length measurements. Small intestinal samples were taken starting from the jejunum (~10 cm distal to the pylorus), and colon samples were taken from the base of the cecum. All tissues were rinsed in PBS and placed into appropriate solutions (liquid nitrogen, RNAlater, or 10% formalin) for later analysis.

### 4.9. Histomorphology

Colon cross sections were fixed in 10% formalin for 24 h and then transferred to 70% ethanol. Tissues were processed overnight using an Excelsior^TM^ ES Tissue Processor (Thermo Scientific; Waltham, MA, USA), embedded vertically in paraffin before being sectioned at 5 µm and stained with hematoxylin and eosin (H&E). Slides were examined blindly under bright field using a Leica DM R microscope (Leica Microsystems; Concord, ON, Canada). Crypt depth and lamina propria width were measured at 100× magnification (field diameter of 0.2 mm) in 10 well-defined crypts in colon cross sections (4 sections for unchallenged, 6 for *C. rodentium*-challenged ProPG-1-treated, 8 for *C. rodentium*-challenged, and 8 for *C. rodentium*-challenged mPG-1-treated groups). Tunica muscularis width was measured from 3 randomly selected regions per cross section at 10× magnification. The average number of neutrophils per high-power field (400× magnification, field diameter of 0.45 mm) was determined from 10 randomly selected regions of the cross section. The average number of goblet cells per 10 well-defined crypts were counted in each cross section from each treatment group. Measurements were taken in a blind fashion using ImageJ Software Version 1.51 (National Institutes of Health, Gaithersburg, MD, USA). Histological damage was assessed single-blindly using methods modified from [62] and [63] (Table S1). Photos were captured with an Olympus BX45 microscope (Olympus America Incorporated; Melville, NY, USA) using cellSens Standard Software Version 1.12 (Olympus America Incorporated; USA)

### 4.10. Real-Time Quantitative PCR

RNA was isolated from frozen colon samples using a Dounce homogenizer for homogenization and the E.Z.N.A^®^ Total RNA Kit II (Omega Bio-Tek; Norcross, GA, USA) according to the manufacturer’s instructions. Total RNA was isolated, treated with RNase-free DNase I (Thermo Fisher Scientific, Waltham, MA, USA), and first-strand complementary DNA (cDNA) was synthesized using 5X All-In-One RT MasterMix (Applied Biological Materials; Richmond, BC, Canada) per manufacturer’s instructions. Samples were run on a 1% agarose gel at 135 V for 40 min to check successful DNase treatment and to assess for RNA degradation. Quantitative RT-PCR (RT-qPCR) was performed using a CFX96 Real-Time PCR Detection System (Bio-Rad; Mississauga, ON, Canada) with SsoAdvanced^TM^ Universal SYBR^®^ Green Supermix (Bio-Rad; Mississauga, ON, Canada). Primer sequences are listed in Table S3. Reactions were performed in duplicate under the following conditions: denaturation at 95 °C for 2 min, three-step amplification including denaturation at 95 °C for 25 s, annealing at 59 °C for 25 s, extension at 70 °C for 15 s, and a subsequent melting curve (65 °C to 95 °C) determination with continuous fluorescence measurement. Mouse primers used were confirmed for a valid efficiency between 90 and 110%. Relative mRNA levels were determined using the comparative Ct method, with glyceraldehyde 3-phosphate dehydrogenase (GAPDH) and β-actin as internal controls.

### 4.11. Statistical Analysis

Statistical analysis was performed using a two-factor analysis of variance (ANOVA) or a one-way repeated measures ANOVA for body weight and DAI over the course of the trials, through either GraphPad Prism Version 7.04 (La Jolla; CA, USA) or SPSS Version 24 software (Armonk; NY, USA). Data sets were analyzed by Tukey’s test for multiple comparisons to determine statistical differences between groups. Data were deemed significant at a *p*-value of * *p* < 0.05, ** *p* < 0.01, *** *p* < 0.001 compared to control. Results are expressed as a mean ± standard error of the mean (SEM).

## Supplementary Materials

Supplementary Materials are available online at https://www.mdpi.com/article/10.3390/ijms22179494/s1.
