# Supplementary material for: Protective and Anti-Inflammatory Effects of Protegrin-1 on Citrobacter rodentium Intestinal Infection in Mice
Source: Int J Mol Sci. 2021 Aug 31;22(17):9494. doi: 10.3390/ijms22179494 (PMC8431371; doi:10.3390/ijms22179494)
Supplement: Supplementary file 1 [file ijms-22-09494-s001.zip › ijms-1332224-supplementary.pdf]

Supplementary materials

Table S1. Definition of categories and general criteria for histomorphological scoring.

CATEGORY	CRITERION	DEFINITION	SCORE VALUE[1]
Inflammatory Cell Infiltrate	Severity	Neutrophil density (per ten 400x high-power fields; field diameter of 0.45 mm):<br>0 = No neutrophils<br>1 = 1 or 2 neutrophils<br>2 = 3 - 5 neutrophils<br>3 = >5 neutrophils	 <br>0<br>1<br>2<br>3
	Extent	Extent of inflammatory cell infiltration:<br>Mucosal<br>Mucosal and submucosal<br>Mucosal, submucosal, and transmural	 0.5<br>1<br>1.5
Epithelial Changes[2]	Hyperplasia	Percent above height of control/baseline crypt (0.2 mm); visible as crypt elongation:<br>No change: 0<br>Mild: 1- 50%<br>Moderate: 51-100%<br>Marked: >100%	 <br>0<br>1<br>2<br>3
	Goblet Cell Count	Change in goblet cell numbers relative to baseline goblet cell numbers per crypt:<br>0 = No change from baseline goblet cell count<br>1 = Increase or decrease of 1-3 goblet cells from baseline<br>2 = Increase or decrease of 4-8 goblet cells from baseline<br>3 = Increase or decrease of 8 or more goblet cells from baseline	 <br>0<br>1<br>2<br>3
	Erosion	Loss of surface epithelium:<br>0 = No pathological changes observed<br>1= Attenuation (gaps of 1-10 epithelial cells/lesion)<br>1.5 = Absence of epithelium (gaps of >10 epithelial cells/lesion)	 0<br>1<br>1.5
	Dysplasia	Ordered arrangement of crypt epithelial cells & crypt epithelial cell nuclei; loss of epithelial polarity<br>Mild: Mostly ordered arrangement, nuclei are in basal location<br>Moderate: Slightly disordered arrangement, some nuclei are near/in apical location<br>Severe: Disordered arrangement, nuclei are in apical location	 <br>0<br>1<br><br>2
Mucosal Architecture	Crypt Loss	Mucosa devoid of crypts	1

[1]Score Value Max = 15 points; [2]From well-oriented samples with longitudinally cut crypts only; Modified from Erben *et al.* (2014) and Bischor *et al.* (2017).

Table S2. Disease Activity Index (DAI) scoring criteria modified from Maxwell *et al.* (2009).

**DISEASE ACTIVITY INDEX (DAI)**			
**Stool Consistency**	**Stool Blood**	**Body Weight Loss (%)[a]**	**Score**
Normal	Normal	0-1 %	0
Moist and Sticky	Brown/Reddish Colour	1-5 %	1
Soft	Visible Blood	5-10 %	2[b]
Diarrhea	Rectal Bleeding	10-20 %[c]	3

[a] $\% \text{ Body Weight Loss} = \frac{(\text{Body Weight on Day X} - \text{Initial Body Weight})}{\text{Initial Body Weight}} \text{ x } 100$

[b] If a mouse scores ≥ 2 in any of the above criteria, supportive therapy must be initiated, and monitoring increased to 4 times per day.

[c] If a mouse scores ≥ 20% body weight loss, it will be euthanized.

Table S3. Primer sequences used for RT-qPCR analysis.

GENE	FORWARD (5' - 3')	REVERSE (5' - 3')	EFFICIENCY (%)
**β-Actin**	CTTCTTTGCAGCTCCTTCGTT	TTCTGACCCATTCCCACCA	96.2
**GAPDH**	CCTCGTCCCGTAGACAAAATG	TGAAGGGGTCGTTGATGGC	92.7
**Muc1**	GGCATTCGGGCTCCTTTCTT	TGGAGTGGTAGTCGATGCTAAG	103.1
**Muc2**	AGGGCTCGGAACTCCAGAAA	CCAGGGAATCGGTAGACATCG	105.5
**Reg3β**	ACTCCCTGAAGAATATACCCTCC	CGCTATTGAGCACAGATACGAG	102.1
**Reg3γ**	ATGCTTCCCCGTATAACCATCA	GGCCATATCTGCATCATACCAG	98.2
**sPLA2**	TGGCTCAATACAGGACCAAGG	GTGGCATCCATAGAAGGCATAG	99.7
**BAX**	TGAAGACAGGGGCCTTTTTG	AATTCGCCGGAGACACTCG	101.2
**BCL3**	CCGGAGGCCCTTTACTACCA	GGAGTAGGGGTGAGTAGGCAG	104.4
**EGR1**	AGACGAGTTATCCCAGCCAAA	GGTCGGAGGATTGGTCATGC	105.7
**TLR6**	TGAGCCAAGACAGAAAACCCA	GGGACATGAGTAAGGTTCCTGTT	100.9
**TLR2**	GCAAACGCTGTTCTGCTCAG	AGGCGTCTCCCTCTATTGTATT	102.0
**SOCS3**	ATGGTCACCCACAGCAAGTTT	TCCAGTAGAATCCGCTCTCCT	105.2
**VCAM-1**	AGTTGGGGATTCGGTTGTTCT	CCCCTCATTCCTTACCACCC	96.5
**HIF1α**	ACCTTCATCGGAAACTCCAAAG	ACTGTTAGGCTCAGGTGAACT	106.5
**Camp**	GCTGTGGCGGTCACTATCAC	TGTCTAGGGACTGCTGGTTGA	102.0
**ANG4**	GGTTGTGATTCCTCCAACTCTG	CTGAAGTTTTCTCCATAAGGGCT	102.1
**IRF7**	ATGCCAATCACTCGAATGAG	TTGTATCGGCCTGTGTGAATG	96.8
**CASP1**	CTTGGAGACATCCTGTCAGGG	AGTCACAAGACCAGGCATATTCT	106.3
